# Contact tracing following measles exposure on three international flights, Germany, 2017

**DOI:** 10.2807/1560-7917.ES.2019.24.19.1800500

**Published:** 2019-05-09

**Authors:** Sebastian Thole, Daniela Kalhoefer, Maria an der Heiden, Doris Nordmann, Inka Daniels-Haardt, Annette Jurke

**Affiliations:** 1 NRW Centre for Health, Department Health Promotion Health Protection, Unit Infectiology and Hygiene, Bochum, Germany; 2 European Programme for Intervention Epidemiology Training (EPIET), European Centre for Disease Prevention and Control (ECDC), Stockholm, Sweden; 3 NRW Centre for Health, Department Health Promotion Health Protection, Unit Infectious Disease Epidemiology, Bochum, Germany; 4 Robert Koch Institute, Department for Infectious Disease Epidemiology, Surveillance Unit, Berlin, Germany; 5 NRW Centre for Health, Department Health Promotion Health Protection, Bochum, Germany

**Keywords:** public health practice, measles, aircraft, communicable disease control, post-exposure prophylaxis, Germany, viral infections, infection control, measles-mumps-rubella vaccine, MMR, vaccines and immunisation

## Abstract

When a person with contagious measles has travelled by aircraft, European guidelines recommend contact tracing of passengers and crew within 5 days of exposure for post-exposure prophylaxis (PEP), and within 12 days of exposure for informing passengers and crew, in order to prevent further transmissions. To be effective, contact tracing requires prompt diagnosis, immediate notification of public health authorities and rapid availability of passenger contact data. We report two events of contact tracing initiated in Germany after two individuals with measles travelled on three international flights. In one event, contact tracing was initiated late because laboratory confirmation of a clinically diagnosed measles case was awaited unnecessarily. Accessing passenger contact data was difficult in both events because of data protection issues with the airline which was not based in Germany. In both events, passengers were not reached in time to provide PEP, and one event resulted in at least two secondary measles cases. As all passengers were reached before the incubation period ended, tertiary cases were most probably prevented. Public health authorities and the transport sector must collaborate to resolve competing legal regulations for infection prevention and data protection, to simplify and accelerate identification of air travellers exposed to communicable diseases.

## Introduction

Measles is an acute, highly infectious viral disease that is usually transmitted by direct contact with infectious droplets. Complications can include ear infections, pneumonia or encephalitis, and are more likely to occur in children younger than 5 years, or adults older than 20 years. Measles cases are infectious from 4 days before to 4 days after the onset of rash [1]. Suspected or confirmed measles cases and laboratory detection of the measles virus in patient material are notifiable to local health authorities (LHA) in Germany under the Infection Protection Act [2]. The two-dose measles vaccination is safe, effective and a well establish standard in immunisation schedules all over Europe.

In 2017, several measles outbreaks, comprising 520 confirmed cases, occurred in North Rhine-Westphalia (NRW), Germany. Measles outbreaks were in that period also reported from other German federal states and several European countries [3].

With thousands of measles cases throughout Europe, it is not surprising that infectious persons travel using public transportation and aircraft. Owing to the infectiousness of measles, transmission in aircraft and in transit areas of airports is possible [4-8].

Contact tracing after an exposure on a flight is not initiated automatically. European risk assessment guidelines for infectious diseases transmitted on aircraft (RAGIDA) require that authorities apply a strong evidence-based rationale before initiating contact tracing [9]. For measles, contact tracing of all flight passengers and attendants is recommended, if “*post-exposure prophylaxis (PEP) can still protect susceptible persons, prevent complications, and limit further transmission*” [9]. Following the RAGIDA guidelines, contact tracing should be performed until Day 5 after exposure with the aim of providing PEP. Priority should be given to children younger than 2 years, pregnant women and immunocompromised contacts. If 6–12 days have passed since the flight, PEP is not likely to prevent illnesses anymore and therefore RAGIDA recommends to only inform passengers and crew and manage cases and susceptible contacts. If more than 12 days have passed, no measures are taken.

Public health authorities that initiate contact tracing may encounter certain difficulties. Measles is likely to be diagnosed only after the index case has left the aircraft and has consulted a healthcare professional. Some days may elapse before the LHA is made aware that a measles case has travelled by aircraft. Therefore, contact tracing involves an immediate request for passenger lists from the airline, as the window for PEP administration is narrow (maximum 72 h for vaccination and 6 days for passive immunisation). According to German recommendations, post-exposure vaccination in adults is recommended for susceptible contacts born after 1970 [10]. Aircraft manifests are not standardised across airlines, and some airlines do not keep passenger lists for more than 48 hours [11]. Data protection issues can hamper the process of exchanging passenger data between airlines and public health authorities [11,12]. Even where contact information is available, some passengers may not be reached in time for PEP [5].

The International Health Regulations (IHR) from 2005 [13] were incorporated into national legislation in Germany in 2007 [14]. The IHR implementing law (*IGV-Durchführungsgesetz,* IGV-DG), entered into force in 2013 and specifies in Article 12(5) (on the basis of article 23 Number 1a IHR) that if a public health authority requests personal contact information of affected passengers or their possible contacts, the airline should provide the data without delay.

As regulated in the German Infection Protection Act, Articles 25 and 27–31 [2], LHAs in Germany are in charge of contact tracing. The overall approach is regulated, but may vary between the 16 federal states of Germany. First informed are most often the LHAs of the district where the case lives or currently resides. This LHA can consult with other LHAs (e.g. the one responsible for the airport where the aircraft landed), the federal state health authority or the Robert Koch Institute (RKI) as the national public health institute. They decide if contact tracing should be initiated and which LHA is in charge and they assign responsibilities in the contact tracing process. The LHA in charge requests passenger data directly from the airline. If data are provided, information about the measles exposure and contact data must be transferred securely to the health authorities of the passengers’ area of residence.

This ensures that (i) outbreak-preventing measures are implemented according to countries’ regulations and recommendations, (ii) language barriers are reduced and (iii) passengers can be provided with contact details of their competent health authority for further queries.

For passengers residing in Germany, the health authority in charge can directly contact other LHAs or the respective federal state health authority. For passengers residing outside of Germany, the LHA may share information with public health authorities abroad via the established international channels: the IHR national focal points (NFP) or through the Early Warning and Response System (EWRS) for countries in the European Union and European Economic Area (EU/EEA). The EWRS also has a mechanism to share personal data under current data protection regulations. As LHAs in Germany do not have access to EWRS, they need to request administrative assistance from the RKI. According to Article 12(7) IGV-DG [14], the RKI is allowed to transfer personal data of passengers if requested by the federal state level (usually by the corresponding ministry of health).

Here we report two events of contact tracing initiated in NRW, Germany, after two individuals with measles travelled on three international flights. We describe the challenges the public health authorities were confronted with during the process and offer suggestions for improving the procedure for future cases.

## Description of two contact tracing events

### Event 1 (single flight)

**Figure 1:**
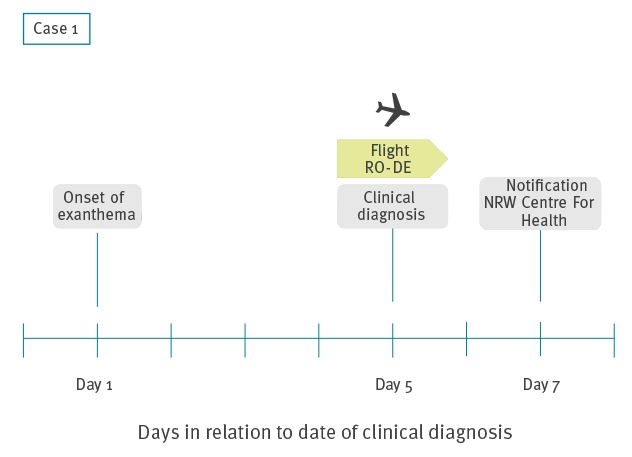
Timeline of events related to measles Case 1, North Rhine-Westphalia, Germany, April 2017

DE: Germany; NRW: North Rhine-Westphalia RO: Romania.

In spring 2017 (Day 7, Friday), the NRW Centre for Health (state health authority; SHA) was informed by LHA 1 about a potentially infectious 2 year-old child with measles who had flown from Romania to NRW 2 days earlier (Day 5) (Case 1, Figure 1). Exanthema started 4 days before the flight and Case 1 was clinically diagnosed with measles directly after the flight, without laboratory confirmation. The child had been visiting family in Romania, where a measles outbreak had been ongoing since January 2016 [15]. One family member in Romania had measles during the visit of Case 1.

### Event 2 (outbound and return flight)

**Figure 2:**
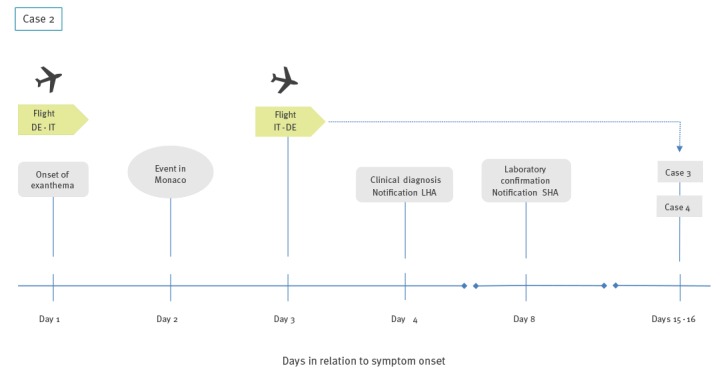
Timeline of events related to measles Case 2, North Rhine-Westphalia, Germany, April 2017

DE: Germany; IT: Italy; LHA: local health authority; SHA: state health authority.

In spring 2017, LHA 2 informed the NRW SHA about a young adult with measles who had flown from NRW to Italy 7 days earlier (Case 2, Day 1, Tuesday, Figure 2). The exanthema started on the day of the outbound flight. From Italy, Case 2 travelled to France by car to attend an event in Monaco the next day. Case 2 returned home via the same route on Day 3. A physician clinically diagnosed measles on Day 4 and notified LHA 2 who initiated laboratory testing. On Day 8, measles was confirmed by IgM antibodies and real-time quantitative PCR. Genotyping was subsequently performed by the German national reference laboratory.

In both events, local and state health authorities decided to initiate contact tracing of flight passengers and crew. The same airline was used for all three flights.

## Contact tracing

### Event 1 (single flight) 

Two days after the flight (Day 7, a Friday) LHA 1 requested data for passengers born after 1970 from the airport administration. The ‘Request Form for Passenger Contact Tracing’ published by the International Air Transport Association (IATA) was used for the request [16]. On the evening of Day 7, the airport provided passenger data in the form of multiple screenshots and one list containing names, birth years and seat numbers. There were single screenshots for all 155 passengers that, however, were of poor image quality and had partly incomplete information (names, addresses, countries, phone numbers, email addresses) or inconsistent content (e.g. non-matching address and international call prefix). From the available information, LHA 1 staff tried during the weekend to determine the places of residence and corresponding public health authorities. The majority of passengers resided in NRW, but there were also passengers from three other German federal states, nine other EU/EEA countries, Serbia and Mexico.

LHA 1 was informed later on Day 7 that the airline maintained a separate register on children younger than 2 years. There were none on the flight in question, but this information was not available until Sunday (Day 9).

For international passengers, the NRW SHA requested contacts of IHR NFPs for the respective countries from the RKI on Monday (Day 10). Contacts of LHAs in NRW were available at the NRW SHA and for other federal states, the respective SHAs were involved. The process of informing all health authorities in Germany and NFPs in other involved countries began on Monday (Day 10). However, transmitting personal passenger data to the health authorities was hampered by the lack of a secure and functional transfer system. Data were transferred by fax, which delayed the process for the international passengers, as some fax numbers for NFPs were unavailable or invalid.

No secondary measles cases were reported that were linked to the flight of Case 1 from Romania to Germany.

### Event 2 (outbound and return flight)

LHA 2 was informed about the possible measles case (Case 2) on Day 4, only 1 day after the return flight, but awaited laboratory confirmation before initiating contact tracing. On Day 8, LHA 2 requested the passenger data from the airline. In agreement with the NRW SHA, passenger data was requested for the return flight only, because 7 days had passed since the outbound flight (Germany to Italy). Contact information for this purpose was not available on the airline’s website, therefore the LHA directed its request to the airline’s service centre, which was difficult and time-consuming.

Even though the index case was very probably highly infectious on both flights, the airline refused to provide passenger data, referring to the data protection law of the country where the airline was based. Furthermore, the airline stated that the list may contain “*passengers who may or may not be linked in any form and may, therefore be irrelevant to the investigation you [the public health authority] are performing*”. However, the airline itself offered to inform all passengers by email. As the window for PEP was closing at the end of that day, LHA 2 decided, in agreement with the NRW SHA and the RKI, not to insist further on the delivery of passenger data and accepted instead the airline’s offer to inform passengers of both flights by email. The text was prepared in English language in cooperation with the airline, LHA 2, the NRW SHA and the RKI. All passengers were advised to monitor themselves for typical signs and symptoms of measles and to contact their local healthcare provider immediately by phone if any symptoms occurred. All 53 LHAs in NRW and all other 15 SHAs in Germany were informed about Case 2, and were requested to report to the NRW SHA any measles cases linked to any of this person’s two flights. The RKI informed other EU/EEA countries via EWRS.

The NRW SHA was informed about two secondary cases linked to the return flight of Case 2. Both had received information from the airline that they might have been exposed to measles. They developed exanthema 15 and 16 days after the flight, respectively (flight from Italy to Germany). One of the secondary cases resided in Rhineland-Palatinate (Case 3) and one in NRW (Case 4). The same genotype was detected in Case 3 and Case 2 (genotype D8, distinct sequence ID 4807), supporting the hypothesis of transmission during the flight. Case 4 was confirmed by antigen testing, but no genotyping was performed.

We did not receive information in either event about whether potentially exposed crew members were informed.

## Discussion

Contact tracing is considered a reasonable intervention, as measles can be transmitted in aircraft and both passengers and cabin crew are at risk. Early contact tracing leading to timely PEP is crucial, but it is unlikely that LHAs are able to perform contact tracing promptly enough. In both events described here, exposed passengers could not be reached in time and secondary measles cases resulted from the second event.

Contact tracing can be substantially delayed by multiple factors, e.g. the lag between clinical diagnosis and notification to LHAs. When laboratory confirmation is sought, several more days may elapse. It is therefore advisable to consider contact tracing immediately upon notification of a clinically diagnosed case. If contact tracing is initiated, reaching the airline to request a passenger list may be difficult and further delay the process, especially when public health authorities need to deal with airline service centres.

The German IHR Implementing Law (IGV-DG (Article 12(5)) explicitly authorises LHAs to request and use personal data of air passengers from airlines in order to prevent the spread of infectious diseases. Provision of passenger data in the context of contact tracing does not interfere with national data protection laws. In fact, facilitation of contact tracing by airlines is reinforced by several international conventions. Article 14 of the Convention on International Civil Aviation (Chicago Convention [17]), for example, states: “*Each contracting State agrees to take effective measures to prevent the spread by means of air navigation of […], and such other communicable diseases […]”*’. The IHR address airlines as well: *“[…] conveyance operators shall facilitate: […] (d) provision of relevant public health information requested by the State Party*” [13]. Similarly, a guideline from the International Civil Aviation Organisation (ICAO) recommends that airlines “*[…] should comply with such a request in a timely manner, and cooperate fully with public health authorities […]*” [18].

Nevertheless, airlines must protect passenger data from unauthorised access. This means that airlines are obliged to verify the request before providing passenger data and ensure secure transfer in accordance with data protection regulations. The process of verification can substantially delay contact tracing activities and may render it impossible to retrieve the data in time to provide exposed passengers with PEP. A possible solution could be to establish dedicated staff at airports who mediate between the involved airline and public health authorities. In addition, technologies for secure online data sharing need to be established that are available to all (local) public health authorities.

Once LHAs obtain passenger information, another time-consuming step is to contact all passengers. For Event 1 described here, sharing information in the network of NRW health authorities and with other German SHAs was relatively easy. Nevertheless, health authorities were reached only on the Monday following the initial information, which was too late for post-exposure vaccination. Reaching the other involved countries was more difficult: ca 1 week passed until all health authorities were informed.

For Event 2, the request for passenger data was unsuccessful. The airline refused to share the data with reference to national data protection regulations. It was the same airline in both events, so it is remarkable that the airline provided passenger data for Event 1 but not Event 2. This may have resulted from the fact that LHA 1 requested passenger data via the airport management, whereas in Event 2, LHA 2 had to contact the airline via their service centre. Since then, the same airline has never shared any passenger data again in two similar events we were involved in.

Nevertheless, the information the airline sent by email was effective and quickly reached all passengers. Earlier distribution of this information could have made a difference, since measles may spread before onset of symptoms and the time frame for PEP is narrow. When Case 3 and Case 4 developed symptoms, they were aware of their exposure to measles on the flight, and this could have been a factor in preventing tertiary cases.

If timely provision of passenger data is not possible, direct communication from the airline to passengers could be a reasonable option, especially under time pressure. The content of the information, however, must be agreed in advance and overseen by the responsible public health authority. Since cabin crew might be at risk of measles infection as well, health authorities should ascertain that information also includes exposed staff. As airlines will probably contact passengers in English language only, not all passengers may understand the content. This is an additional advantage of making passenger data available to the public health authorities who may want to communicate directly with passengers, even if an email to the passengers has been sent out by the airline.

According to our experiences, contact tracing takes more time than expected according to RAGIDA. It may therefore be worth considering a revision of the guidelines on measles contact tracing. RAGIDA recommends performing contact tracing up to 5 days after the flight in question, assuming that at least 1 day is needed to get the data, reach the passengers or the responsible public health authorities and provide the passengers with PEP (passive immunisation is possible for up to 6 days after exposure). This is hardly feasible, especially during the weekend. However, even if it seems impossible to perform contact tracing fast enough to provide PEP and prevent secondary cases, information of passengers, crew and public health authorities after this time frame can still prevent tertiary measles cases. Our recommendations on efficient flight-related contact tracing upon notification of a measles case are listed in the Box.

Box **Recommendations for flight-related contact tracing upon notification of a measles case**

PHAs should consider contact tracing immediately upon notification of a measles case.
If PHAs decide to perform contact tracing, they should always request passenger data from the airline with the aim to provide timely PEP and to prevent tertiary cases.
PHAs should prioritise children younger than 2 years, pregnant women and immunocompromised contacts, if this information is available.
PHAs can consider contacting airport management if airline telephone service centres are not available.
PHAs can ask airlines to inform exposed passengers and crew by email as an additional or alternative means of communication, if the airline does not provide passenger data in time.
PHAs should approve the content of health-related information sent out by an airline. Ideally, templates would be available in multiple languages and advocated by international organisations like WHO and IATA.
Dedicated contact persons at airports could mediate between airlines and PHAs and facilitate future contact tracing.
Technologies for secure online data exchange need to be established and made available to PHAs.

IATA: International Air Transport Association; PHA: public health authority; PEP: post-exposure prophylaxis; WHO: World Health Organization.

As an interim solution, public health authorities should have information for potential contact persons at hand in order to provide adequate information. Ideally, such information would be available in multiple languages (e.g. the six official United Nations languages). Like the World Health Organization (WHO) passenger locator form, such documents could be provided via existing collaborations between the WHO, the ICAO and the IATA, such as the Collaborative Arrangement for the Prevention and management of public health events in Civil Aviation (CAPSCA) [19].

Public health authorities and airlines have the same goal: “*to ensure safe and healthy air travel for passengers which are travelers and crew members”* [20]. It is the role and responsibility of public health authorities to assess whether contact tracing should be initiated and which persons are to be defined at risk of exposure or infection. The statement from the airline that some passengers might be irrelevant to the investigation clearly indicates an unnecessary conflict regarding the roles. 

## Conclusions

Collaboration between public health authorities and airlines is inevitable, and providing passengers with health information by email has proved to be practical. Nevertheless, the responsibility and expertise of public health authorities is clearly defined and should not be abandoned.

Health authorities have to be notified immediately when a possible measles case has travelled by aircraft. Tracing of contacts must be started as soon as possible to be effective. Consequently, obtaining passenger contact information from airlines needs to be improved. Technical means that comply with data protection regulations should be developed and deployed to enable secure data sharing between airlines and health authorities. Intersectoral efforts on regional and international level are needed to improve the current situation, raise awareness among all stakeholders involved in contact tracing and facilitate the process of contact tracing in a way that public health remains a public responsibility.

## References

[1] World Health Organization (WHO). Measles. Fact sheet Geneva: WHO; 2018. Available from: www.who.int/mediacentre/factsheets/fs286/en/

[2] Gesetz zur Verhütung und Bekämpfung von Infektionskrankheiten beim Menschen (Infektionsschutzgesetz - IfSG). [Act for the prevention and control of human infectious diseases (Protection against infection act)]. Berlin: Bundesministerium der Justiz und für Verbraucherschutz. 2018. Bundesgesetzblatt Part I, 20 Jul 2000, pp. 1045-77. Zuletzt geändert durch Artikel 6 des Gesetzes vom 11.12.2018 (BGBl. I S. 2394). German. Available from: https://www.gesetze-im-internet.de/ifsg/IfSG.pdf

[3] European Centre for Disease Prevention and Control (ECDC). Measles and rubella surveillance 2017. Stockholm: ECDC; 2018. Available from: https://ecdc.europa.eu/sites/portal/files/documents/Measles-and-Rubella-Surveillance-2017.pdf

[4] Coleman KP Markey PG Measles transmission in immunized and partially immunized air travellers. Epidemiol Infect. 2010;138(7):1012-5. 10.1017/S0950268809991129 19878613

[5] Cotter S, O’Flanagan D, Cooney F, Thornton L, McKeown P. Probable measles transmission during transatlantic travel. Epi-Insight. 2010;11(8). Available from: http://ndsc.newsweaver.ie/epiinsight/ghclnh7rdptt6pex6po7cd

[6] Edelson PJ Patterns of measles transmission among airplane travelers. Travel Med Infect Dis. 2012;10(5-6):230-5. 10.1016/j.tmaid.2012.10.003 23127863

[7] Mangili A Gendreau MA Transmission of infectious diseases during commercial air travel. Lancet. 2005;365(9463):989-96. 10.1016/S0140-6736(05)71089-8 15767002 PMC7134995

[8] Nic Lochlainn L Mandal S de Sousa R Paranthaman K van Binnendijk R Ramsay M A unique measles B3 cluster in the United Kingdom and the Netherlands linked to air travel and transit at a large international airport, February to April 2014. Euro Surveill. 2016;21(13):30177. 10.2807/1560-7917.ES.2016.21.13.30177 27074646

[9] European Centre for Disease Prevention and Control (ECDC). Risk assessment guidelines for diseases transmitted on aircraft. 2nd ed. Stockholm: ECDC; 2010. Available from: https://ecdc.europa.eu/sites/portal/files/media/en/publications/Publications/1012_GUI_RAGIDA_2.pdf

[10] Robert Koch Institute. Empfehlungen der Ständigen Impfkommission (STIKO) am Robert Koch-Institut –2017/2018. [Recommendations of the Standing Committee on Vaccination at the Robert Koch Institute – 2017/2018]. Epidemiologisches Bulletin. 2017;34. German. Available from: https://www.rki.de/DE/Content/Infekt/EpidBull/Archiv/2017/Ausgaben/34_17.pdf;jsessionid=F7888EF84FE57273A69E6E10D746B3F0.1_cid363?__blob=publicationFile

[11] Leitmeyer K European risk assessment guidance for infectious diseases transmitted on aircraft--the RAGIDA project. Euro Surveill. 2011;16(16):19845. 21527131

[12] Swaan CM Appels R Kretzschmar ME van Steenbergen JE Timeliness of contact tracing among flight passengers for influenza A/H1N1 2009. BMC Infect Dis. 2011;11(1):355. 10.1186/1471-2334-11-355 22204494 PMC3265549

[13] World Health Organization (WHO). International Health Regulations. 3rd ed. Geneva: WHO; 2005. Available from: https://apps.who.int/iris/bitstream/handle/10665/246107/9789241580496-eng.pdf?sequence=1

[14] Gesetz zur Durchführung der Internationalen Gesundheitsvorschriften 2005 (IGV-Durchführungsgesetz - IGV-DG). [Act to implement the International Health Regulations (2005)]. Berlin: Bundesministerium der Justiz und für Verbraucherschutz. Bundesgesetzblatt Part I, 28 Mar 2013, pp. 566-84. German. Available from: http://www.ilo.org/dyn/natlex/docs/ELECTRONIC/95783/112899/F-216424569/bgbl113s0566_20818.pdf

[15] European Centre for Disease Prevention and Control (ECDC). Ongoing outbreak of measles in Romania, risk of spread and epidemiological situation in EU/EEA countries - 3 March 2017. Stockholm: ECDC; 2017. Available from: https://ecdc.europa.eu/sites/portal/files/media/en/publications/Publications/27-02-2017-RRA-Measles-Romania%2C%20European%20Union%20countries.pdf

[16] International Air Transport Association (IATA). Request Form for Passenger Contact Tracing. Montreal: IATA. [Accessed: 5 Sep 2018]. Available from: www.iata.org/whatwedo/safety/health/Documents/request-form-passenger-contact-tracing.pdf

[17] International Civil Aviation Organization (ICAO). Convention on international civil aviation. Montreal: ICAO; 2006. Available from: https://www.icao.int/publications/Documents/7300_9ed.pdf

[18] International Civil Aviation Organization (ICAO). Managing communicable disease in aviation. Montreal: ICAO. [Accessed: 5 Sep 2018]. Available from: www.icao.int/safety/aviation-medicine/Pages/healthrisks.aspx

[19] Collaborative Arrangement for the Prevention and Management of Public Health Events in Civil Aviation (CAPSCA). [Accessed: 12 Dec 2018]. Available from: www.capsca.org/

[20] AIRSAN. Contact tracing – collaboration between the public health and the aviation sector. AIRSAN; 2015. Available from: www.airsan.eu/Portals/0/docs/AIRSAN_Guidance%20Document_Contact%20Tracing_May2015.pdf

